# Pneumonia in Geriatric Patients and Prediction of Mortality Based on the Pneumonia Severity Index (PSI), CURB-65, Frailty Index (FI), and FI-Lab21 Scores

**DOI:** 10.7759/cureus.61719

**Published:** 2024-06-05

**Authors:** Firdaus Jabeen, Ajay Mishra, Saboor Mateen, Ankit Maharaj, Rishabh Kapoor, Syed Faraz Abbas, Shahedullah Khan, Abhinaya Gupta

**Affiliations:** 1 Internal Medicine, Era's Lucknow Medical College and Hospital, Lucknow, IND

**Keywords:** geriatric, pneumonia severity index, frailty index, curb-65, community-acquired pneumonia

## Abstract

Background

Elderly individuals have higher rates of morbidity, death, and financial burden due to community-acquired pneumonia (CAP).

Objectives

The study aimed to assess the outcomes of geriatric pneumonia patients and the prediction of mortality based on the pneumonia severity index (PSI), CURB-65 (confusion, urea, respiratory rate, blood pressure, and 65-year-old score), frailty index (frailty index), and FI-Lab21 (21-item frailty index based on laboratory) scores.

Methods

A prospective observational study was conducted on 100 elderly patients (≥ 65 years) with CAP. PSI, CURB-65, FI, and FI-Lab21 scores were determined. The outcome measures were 30-day mortality and the risk factors of mortality. The mortality predictive value of scores were compared.

Results

The mean age of the study subjects was 72.14 ± 6.1 years. Specifically, 76 (76%) were male, and 24 (24%) were females. During the follow-up, there was a 30-day mortality rate of 57%. On performing multivariate regression, the PSI score and severely frail were significant independent risk factors of mortality, with an odds ratio of 1.046 and 52.213, respectively. Area under the ROC curve (AUC) showed that the performance of the PSI score (AUC: 0.952; 95% CI: 0.910-0.994), CURB-65 score (AUC: 0.936; 95% CI: 0.893-0.978), and severely frail (AUC: 0.907; 95% CI: 0.851-0.962) was outstanding, while FI-Lab21 (AUC: 0.515; 95% CI: 0.400-0.631) was non-significant. Among all the parameters, the PSI score was the best predictor of mortality at the cutoff points of >121 with a diagnostic accuracy of 92%.

Conclusion

CAP in the elderly carries a high mortality rate. Out of PSI, CURB-65, FI, and FI-Lab21 scores, the PSI holds the best predicting ability for mortality.

## Introduction

Population aging (referring to the demographic shift towards an increasing proportion of older adults) [1] has brought on a range of implications for health [2]. Projections suggest that, by 2050, approximately 21% of the global population will be classified as elderly [3].

As individuals age, their risk of developing community-acquired pneumonia (CAP) rises, significantly affecting morbidity and mortality among the elderly [4]. The reported incidence rates of CAP in different populations vary between 1.3 and 11.6 cases per 1,000 inhabitant-years, with the highest rates in elderly adults (13-15 cases per 1,000 inhabitant-years) [5].

Unlike in younger adults, pneumonia in older adults presents with unclear onset, atypical clinical signs, numerous complications, and a complex underlying cause due to age-related changes and existing medical conditions. These factors contribute to delays in diagnosis and treatment, ultimately leading to higher short-term and long-term mortality rates in this population [6].

Since the elderly population is frail, defined by a decline in biological reserves, the breakdown of homeostatic mechanisms, and heightened susceptibility to various challenges or adversities, the prognosis among them remains adverse in relation to the severity of the disease. Predicting mortality risk among them becomes crucial for risk stratification and improving outcomes [7,8].

Common tools such as the pneumonia severity index (PSI), frailty index (FI) [9,10], 21-item FI based on laboratory (FI-Lab21) values, and CURB-65 (confusion, urea, respiratory rate, blood pressure, and 65-year-old score) [8] are frequently utilized for this purpose. However, these tools may have limitations when applied to older patients due to factors such as comorbidities, age-related physiological changes, and practical application difficulties [7,8].

Studies remain sparse in assessing which of these scores holds superiority over another for predicting mortality risk in elderly patients with pneumonia. Thus, we conducted this study wherein all scores were included, and we assessed the outcomes in terms of these scores among aged patients presenting with pneumonia.

## Materials and methods

A prospective observational study was conducted on 100 elderly patients who presented to the OPD with CAP. Ethical committee clearance was obtained before initiating the study, and informed consent was obtained from the patients before they were enrolled. The study was conducted over a period of 24 months from April 2022 to March 2024. The sample size calculation was based on the study by Zan et al. [9]. In their research, they found that the AUC values for FI-Lab, PSI, and CURB-65 in predicting mortality were 0.783, 0.812, and 0.799, respectively. With that reference value, along with a significance level of 5% and a specified δ of 0.065, the calculated sample size was 99 patients.

Selection criteria

Patients aged ≥65 years hospitalized with CAP during the study period were included. The exclusion criteria were secondary causes of pneumonia, such as active tuberculosis, bronchiectasis, cystic pulmonary fibrosis, lung abscesses, Pneumocystis carinii pneumonia, radiation pneumonia, or patients with incomplete data (including clinical information, auxiliary examination results, etc.).

Methodology

The data of patients on demographics, comorbidities, and vital signs were collected on a pre-designed questionnaire. Clinical presentation was noted, which included fever, cough with or without sputum production, dyspnea, fatigue, anorexia, chest discomfort, delirium, lethargy, and a history of falls. Pneumonia was diagnosed based on clinical and laboratory findings such as arterial blood gas (ABG), complete blood count (CBC), electrolytes (sodium, potassium, chloride, calcium, magnesium, phosphate), blood urea nitrogen (BUN), procalcitonin (PCT), C-reactive protein (CRP), blood culture, and sputum culture. Chest X-ray was done on all patients.

Standards and criteria

An FI [11] was determined by assessing 50 factors at baseline, including 25 comorbidities, taking more than five prescription drugs, self-reported ability to perform 21 activities, experiencing weight loss of over 5 kg in the past year, having a body mass index (BMI) below 21, and having a serum albumin level below 3.5 g/L. Using the FI scale, which ranges from 0 to 1, patients were categorized as robust (<0.15), pre-frail (range: 0.15-0.24), mildly to moderately frail (range: 0.25-0.44), or severely frail (≥0.45) [12]. Based on admission information, the PSI [13] (range: 0-395) and CURB-65 [12] (range: 0-5) scores were calculated. The PSI is composed of 20 clinical, exploratory, and analytical variables, whereas the CURB-65 assesses the level of consciousness, blood urea, systolic blood pressure, respiratory rate, and age of patients. The FI-Lab21 score, a 21-item FI based on laboratory blood and urine tests, was also assessed [14]. The patients were followed up for discharge and mortality up to 30 days. Any patient discharged before 30 days was followed up telephonically until 30 days. The outcome measures were 30-day mortality and risk factors of mortality.

Statistical analysis

Quantitative data, assuming a normal distribution, were expressed as means with standard deviations, while categorical variables were presented as counts and percentages. Tests included the independent t-test for quantitative variables and the chi-square test (or Fisher’s exact test, if necessary) for qualitative variables. Receiver operating characteristic (ROC) analysis was applied to determine the predictive values for mortality using the PSI score, CURB-65 score, severely frail, and FI-Lab21, with the DeLong test for comparative analysis. Multivariate logistic regression identified significant mortality risk factors. Data were initially entered in Microsoft Excel and analyzed using Statistical Product and Service Solutions (SPSS, version 25.0; IBM SPSS Statistics for Windows, Armonk) software. A p-value of <0.05 indicated statistical significance across all analyses.

## Results

**Figure 1:**
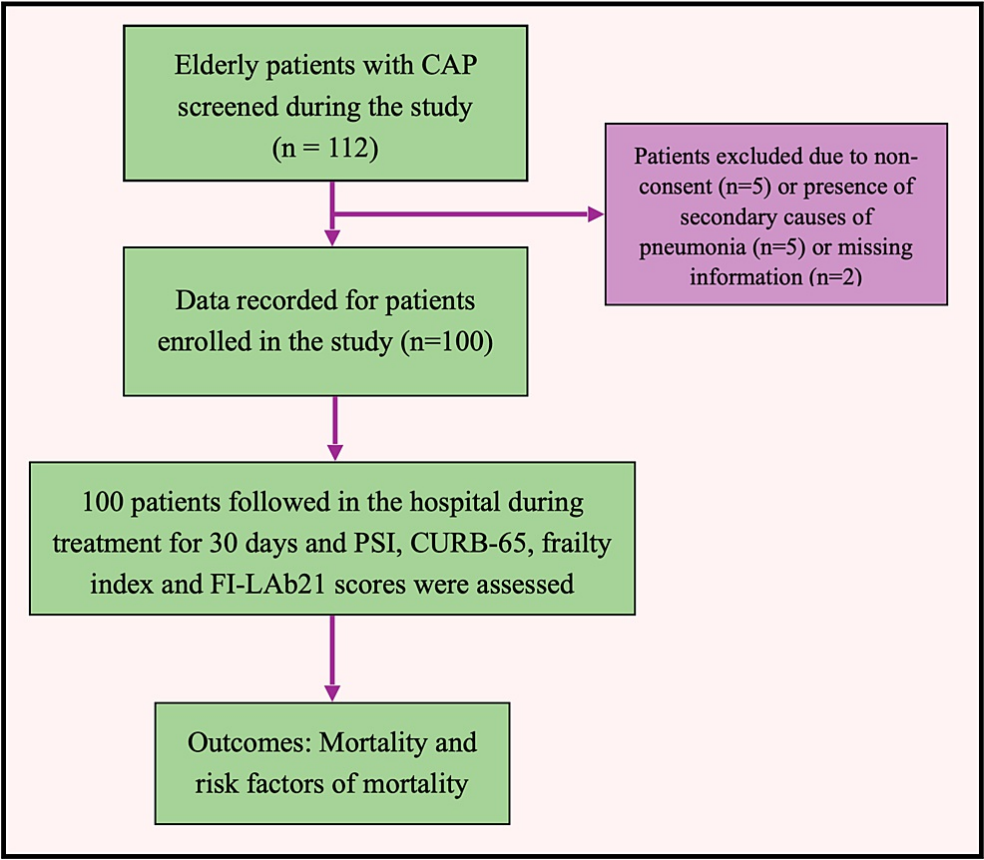
Study flow diagram CAP: Community-acquired pneumonia; CURB-65: Confusion, urea, respiratory rate, blood pressure, and 65-years-old score; FI-Lab21: Frailty index associated with laboratory values; PSI: Pneumonia severity index

During the study period, 112 patients were found eligible, of which five were excluded because of no consent, five were excluded because they had secondary causes of pneumonia, and two were excluded because of missing data. Thus, 100 patients were evaluated for the study (Figure 1).

Demographic characteristics

The mean age of the study subjects was 72.14 ± 6.1 years. Specifically, 76 (76%) were male, and 24 (24%) were females. Regarding frailty status, 51% (n=51) of cases were severely frail, 29% (n=29) were moderately frail, and 20% (n=20) were classified as robust. Hypertension was present in 62% (n=62) of cases, type II diabetes mellitus in 55% (n=55), confusion in 45%, a history of congestive heart failure (CHF) in 16%, cerebrovascular history in 5%, and a history of renal disease in 4% patients. The mean values of the PSI score, CURB-65 score, and FI-Lab21 score were 133.56 ± 41.03, 2.75 ± 1.29, and 0.4 ± 0.14, respectively (Table 1).

**Table 1:** Demographic characteristics of enrolled patients PSI: Pneumonia severity index; FI-Lab21: Frailty index associated with laboratory values; CURB-65: Confusion, urea, respiratory rate, blood pressure, and 65 years old score; CHF: Congestive heart failure

Patient characteristics	Number (Percentage %)	Mean ± SD	Median (25th-75th percentile)	Range
Age (years)	N/A	72.14 ± 6.1	70 (67-75)	65-90
Gender				
Female	24 (24%)	N/A	N/A	N/A
Male	76 (76%)			
FI				
Robust	20 (20%)	N/A	N/A	N/A
Moderate frail	29 (29%)			
Severely frail	51 (51%)			
Hypertension	62 (62%)	N/A	N/A	N/A
Type II diabetes mellitus	55 (55%)	N/A	N/A	N/A
History of CHF	16 (16%)	N/A	N/A	N/A
History of cerebrovascular disease	5 (5%)	N/A	N/A	N/A
History of renal disease	4 (4%)	N/A	N/A	N/A
Confusion	45 (45%)	N/A	N/A	N/A
PSI score	N/A	133.56 ± 41.03	126 (99.75-173.5)	67-235
CURB-65 score	N/A	2.75 ± 1.29	3 (2-4)	1-5
FI-Lab21	N/A	0.4 ± 0.14	0.38 (0.31-0.46)	0.1-0.93

Outcomes

During the follow-up, there was a 30-day mortality rate of 57%, while 43% of cases were discharged. Compared to survivors, non-survivors were significantly more severely frail (85.96% vs. 4.65%, p<0.0001); had significantly more confusion (75.44% vs. 4.65%, p<0.0001); had significantly higher ages (73.68 ± 6.6 vs. 70.09 ± 4.79 years, p=0.002); had more PSI scores (160.7 ± 30.78 vs. 97.58 ± 19.52, p<0.0001); and had higher CURB-65 scores (3.6 ± 0.94 vs. 1.63 ± 0.69, p<0.0001), but they had similar FI-Lab21 (0.4 ± 0.13 vs. 0.39 ± 0.15, p=0.812) and similar gender distribution and comorbidities (p>0.05, Table 2).

**Table 2:** Association of patient characteristics with outcomes ‡ Independent t-test, * Fisher's exact test, † Chi-square CURB-65: Confusion, urea, respiratory rate, blood pressure, and 65 years old score; FI: Frailty index; FI-Lab21: Frailty index associated with laboratory values; PSI: Pneumonia severity index

Patient characteristics	Discharged (n=43)	Expired (n=57)	Total	P value
Age(years)	70.09 ± 4.79	73.68 ± 6.6	72.14 ± 6.13	0.002^‡^
Gender				
Female	14 (32.56%)	13 (22.81%)	27 (27%)	0.277^†^
Male	29 (67.44%)	44 (77.19%)	73 (73%)	
FI				
Robust	20 (46.51%)	0 (0%)	20 (20%)	<.0001^†^
Moderate frail	21 (48.84%)	8 (14.04%)	29 (29%)	
Severely frail	2 (4.65%)	49 (85.96%)	51 (51%)	
Hypertension	28 (65.12%)	34 (59.65%)	62 (62%)	0.577^†^
Type II diabetes mellitus	19 (44.19%)	36 (63.16%)	55 (55%)	0.059^†^
History of CHF	7 (16.28%)	9 (15.79%)	16 (16%)	0.947^†^
History of cerebrovascular disease	2 (4.65%)	3 (5.26%)	5 (5%)	1^*^
History of renal disease	1 (2.33%)	3 (5.26%)	4 (4%)	0.632^*^
Confusion	2 (4.65%)	43 (75.44%)	45 (45%)	<.0001^*^
PSI score	97.58 ± 19.52	160.7 ± 30.78	133.56 ± 41.03	<.0001^‡^
CURB-65 score	1.63 ± 0.69	3.6 ± 0.94	2.75 ± 1.29	<.0001^‡^
FI-Lab21	0.39 ± 0.15	0.4 ± 0.13	0.4 ± 0.14	0.812^‡^

Risk factors of mortality

On performing multivariate regression, the PSI score and severely frail were significant independent risk factors of mortality, with odds ratios of 1.046 and 52.213, respectively, after adjusting for confounding factors (Table 3).

**Table 3:** Multivariate logistic regression to find out the significant risk factors of mortality PSI: Pneumonia severity index; CURB-65: confusion, urea, respiratory rate, blood pressure, and 65 years old score

Variables	Beta coefficient	Standard error	P value	Odds ratio	Odds ratio Lower bound (95%)	Odds ratio upper bound (95%)
Age (years)	-0.104	0.084	0.216	0.901	0.764	1.063
PSI score	0.045	0.018	0.014	1.046	1.009	1.085
CURB-65 score	0.631	0.522	0.227	1.879	0.676	5.224
Robust/moderate frail/severely frail						
Robust	N/A	N/A	N/A	1.000	N/A	N/A
Moderate frail	2.026	1.606	0.207	7.582	0.326	176.618
Severely frail	3.955	1.937	0.041	52.213	1.173	2323.655
Confusion	0.001	1.236	0.999	1.001	0.089	11.284

Mortality prediction of scores

ROC curves above the diagonal line are considered to have a reasonable discriminating ability to predict mortality. Interpretation of the AUC showed that the performance of the PSI score (AUC: 0.952; 95% CI: 0.910-0.994), CURB-65 score (AUC: 0.936; 95% CI: 0.893-0.978), and severely frail (AUC: 0.907; 95% CI: 0.851-0.962) was outstanding. On the other hand, the discriminatory power of FI-Lab21 (AUC: 0.515; 95% CI: 0.400-0.631) was non-significant. FI-Lab21 had a sensitivity of 89.47%, followed by the PSI score (87.72%), severely frail (85.96%), and CURB-65 score (84.21%) for predicting mortality. The PSI score had a specificity of 97.67%, followed by severely frail (95.35%), CURB-65 score (93.02%), and FI-Lab21 (18.60%) for predicting mortality. Among all the parameters, the PSI score was the best predictor of mortality at cutoff points of >121 with an AUC of 0.952 for correctly predicting mortality (Table 4).

**Table 4:** Receiver operating characteristic of PSI score, CURB-65 score, severely frail, and FI-Lab21 for predicting mortality PPV: Positive predictive value; NPV: Negative predictive value

Variables	PSI score	CURB-65 score	Severely frail	FI-Lab21
Area under the ROC curve (AUC)	0.952	0.936	0.907	0.515
Standard error	0.0215	0.0216	0.0283	0.0591
95% Confidence interval	0.910-0.994	0.893-0.978	0.851-0.962	0.400-0.631
P value	<0.0001	<0.0001	<0.0001	0.7956
Cutoff	>121	>2	-	>0.26
Sensitivity (95% CI)	87.72% (76.3-94.9%)	84.21% (72.1-92.5%)	85.96% (74.2-93.7%)	89.47% (78.5-96.0%)
Specificity (95% CI)	97.67% (87.7-99.9%)	93.02% (80.9-98.5%)	95.35% (84.2-99.4%)	18.6% (8.4-33.4%)
PPV (95% CI)	98% (89.6-100.0%)	94.1% (83.8-98.8%)	96.1% (86.5-99.5%)	59.3% (48.2-69.8%)
NPV (95% CI)	85.7% (72.8-94.1%)	81.6% (68.0-91.2%)	83.7%	57.1% (28.9-82.3%)
Diagnostic accuracy	92%	88%	90%	59%

**Figure 2:**
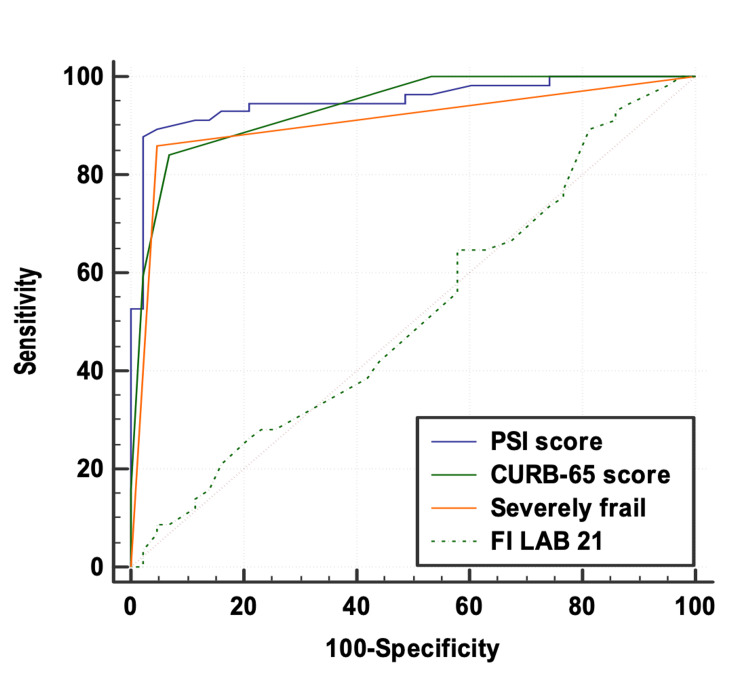
Comparison of the area under the curve of the PSI score, CURB-65 score, severely frail, and FI-Lab21 for predicting mortality (DeLong test) PSI: Pneumonia severity index; FI-Lab21: Frailty index associated with laboratory values; CURB-65: confusion, urea, respiratory rate, blood pressure, and 65 years old score

No significant difference was seen in the AUC of the PSI score, CURB-65 score, and severely frail for predicting mortality. However, FI-Lab21 had a significantly lower AUC as compared to the PSI score, CURB-65 score, and severely frail for predicting mortality (p<0.0001; Figure 2).

## Discussion

The lethality of pneumonia tends to increase with age, especially in patients over 65 years. Specifically, age ≥ 90 years is reported to be markedly associated with mortality due to pneumonia [15,16]. Our study observed a high mortality rate in elderly CAP patients, wherein 57% of patients died and 43% survived. In comparison, Nascè et al. [17] found that, out of 200 suspected pneumonia patients, 60 died, resulting in a mortality rate of 30%. In the study by Zan et al. [9], out of 495 participants, 74 (15%) died and 421 (85%) survived. In Park et al. [18], out of 190 patients, 53 (27.9%) died and 137 (72.1%) survived. Baek et al. [19] reported that, out of 160 patients with severe pneumonia, 40 (25%) died. In a study by Ilg et al. [20], on 2,322 patients suspected of pneumonia, 97 (4.2%) died in hospital. Overall, the mortality rate has been lower in previous studies compared to our study. These values may differ based on the populations studied, hospital facilities, and the severity of the disease. Such a high mortality rate in the elderly can be attributed to frailty or various comorbidities, such as hypertension, diabetes mellitus, CHF, cerebrovascular disease, and renal disease, as observed in our study. Studies have consistently shown the association of factors such as age (p<0.01), being critically ill (p<0.01), and the presence of comorbidities (p<0.01) with mortality [9,17]. Studies have also found lower BMI and hypoalbuminemia as independent prognostic factors associated with five-year mortality [18,21], though these factors were not assessed in the present study.

Similarly, when we compared the factors among those who died and those who survived, we found that higher age and severe frailty were significantly associated with mortality. However, after adjusting for confounding factors, multivariate logistic regression showed that age, gender, and comorbidities were not significantly associated with mortality. However, patients who were severely frail showed poor outcomes, with an odds ratio of 52.213 (p=0.041), which was in line with the studies by Zan et al. [9] and Park et al. [18]. Among the various scores assessed, only the PSI score was found to be a significant independent risk factor for mortality, with an odds ratio of 1.046 (95% CI: 1.009-1.085, p=0.014). In terms of predicting mortality, the PSI score showed the highest accuracy in predicting mortality (92% at a cutoff of >121). In comparison, CURB-65 had an accuracy of 88% at a cutoff of >2, severe frailty had a diagnostic accuracy of 90%, and the FI-Lab21 index had a 59.6% accuracy for predicting mortality with a cutoff value of >0.296.

This was in accordance with the findings by Zan et al. [9], who found that the diagnostic accuracy in predicting 30-day mortality was highest for PSI (AUC: 0.812), followed by FI-Lab21 (AUC: 0.783) and CURB-65 (AUC: 0.799, p<0.01). Similarly, Park et al. [18] found that PSI had the highest accuracy in predicting six-month mortality, followed by FI-Lab21 and CURB-65. The C-statistics for the PSI score was 0.71, for FI-Lab21 was 0.69, and for CURB-65 was 0.62. In contrast, Nascè et al. [17] did not find PSI or CURB-65 scores to be significant predictors of mortality. Rather, they found higher age, lower BMI, and scores assessing comorbidities and malnutrition to be important predictors of one-year mortality. Similarly, Baek et al. [19] found that the CURB-65 and PSI scores did not perform well in predicting outcomes for older patients with pneumonia, as the AUC for predicting mortality in pneumonia was 0.61 for the CURB-65 score and 0.52 for the PSI score. The reasons attributed were a higher proportion of comorbidities in the patients, more nursing home patients included who may have compromised immunity, different exposure to microbiological organisms, and outcome measures of only in-hospital mortality rather than 30-day mortality.

Overall, the scores remain useful for predicting mortality, and the PSI was found to be superior to others. This may be because the PSI has been developed using large datasets from diverse patient populations, which enhances its generalizability. However, it must be stressed that, while the PSI is effective in predicting outcomes, its comprehensive assessment often requires various diagnostic tests such as chest radiographs and arterial blood gas analysis. In resource-poor settings, obtaining these tests can be challenging, leading to potential limitations in using the PSI for prognostication in such contexts [9]. In those cases, other scores such as CURB-65 and FI may become handy, as they carry statistically comparable diagnostic accuracy to the PSI. A recent study by Zan et al. [9] has further shown that the FI-Lab21 score can be judiciously used by combining it with the PSI and CURB-65, which may increase its AUC from 0.812 to 0.85 and from 0.799 to 0.839, respectively.

## Conclusions

To conclude, the PSI, along with CURB-65 and FI-Lab21, scores exhibited correlations with 30-day in-hospital mortality among elderly patients with CAP. The PSI score demonstrated better predictive ability than those of CURB-65 and FI-Lab21 in predicting mortality. It appears to be a simple, efficient, easily accessible, and objective tool that can assist clinicians in promptly stratifying older CAP patients. Identifying the risk of mortality in elderly patients with CAP early upon admission would enable clinicians to intervene promptly during the hospital stay. This can help prevent functional decline and allow for the development of a suitable rehabilitation plan to restore function after discharge.
